# Intravenous Pamidronate for Refractory Lymphedema

**Published:** 2011-04-01

**Authors:** A A Beigi, A M Sadeghi, H Masoudpour, S Shirazinejad, P Mottaghi

**Affiliations:** 1 Department of Vascular Surgery, Isfahan University of Medical Sciences, Alzahra Hospital, Isfahan, Iran; 2 Department of Surgery, Kashan University of Medical Sciences, Kashan, Iran; 3 Department of Surgery,Isfahan University of Medical Sciences, Alzahra HospitalIsfahan, Isfahan, Iran; 4 Department of Radiology,Isfahan University of Medical Sciences, Alzahra HospitalIsfahan, Isfahan, Iran; 5 Department of Internal Medicine, Isfahan University of Medical Sciences, Alzahra Hospital, Isfahan, Iran

**Keywords:** Bisphosphonate, Pamidronate, Refractory lymphedema

## Abstract

**Background:**

Based on beneficial reports of pamidronate use for reflex sympathetic dystrophy in reduction of pain and swelling, this drug can be studied as a novel treatment for refractory lymphedema. This study aims to determine the effectiveness of pamidronate on lymphedema and its possible side effects.

**Methods:**

Twelve cases of lower limb refractory lymphedema were enrolled. They received intravenous pamidronate monthly for 3 consecutive months and were followed by measuring any discomfort with visual analog scale (VAS) and physician global assessment, based on objective signs of limb volume and circumference.

**Results:**

The limb volume, circumference, and satisfaction of the patients improved significantly.

**Conclusion:**

Pamidronate when is added to conservative treatments may reduce lymphedema and improve the patient’s comfort.

## Introduction

Lymphedema is generally defined as an excessive regional accumulation of protein-rich fluid in the extravascular interstitial spaces as a consequence of impaired lymphatic drainage,[1][2][3] and is a frequent but often neglected sequel of cancer treatment.[4][5][6][7] The lymphedema in extremities may cause great discomfort for the patient due to accumulation of large amounts of fluid causing restriction in joint motion.[4][5][6] The standard treatment for this edema has been the use of bandaging (stocking, sleeves), compression garment, exercise, manual lymphatic drainage, and skin care.[1][3][8][9][10] Nevertheless, some of patients with persistent or reluctant extremity lymphedema need an additional treatment measure. [10][11][12][13][14][15][16][17][18] The most commonly used drugs in lymphedema are benzopyrones,[19][20] but a rate of 6% hepatotoxicity reported in an American multicenter study caused benzopyrone preparations not to be recommended for a long term therapy.[21] Other described drugs such as corticosteroids and diuretics are also not recommended for the treatment of lymphedema.[1][8][21] Effects of pamidronate on bone physiology and in reflex sympathetic dystrophy have been previously investigated. It has an osteoclastic inhibitory effect and has the ability to inhibit afferent nerve fibers,[22][23][24][25] but little is known about the basic pharmacologic mechanisms of pamidronate and its effects on reduction of edema. After recent beneficial effects using pamidronate in treatment of reflex sympathetic dystrophy with a low rate of side effects, pamidronate can be a novel option for therapy of recalcitrant lymphedema that is not investigated before. This study investigates the efficacy and possible side effects of pamidronate on lymphedema.

## Materials and Methods 

Between January and June 2007, 12 consecutive patients with at least 6 months persistent or progressive lymphedema, unresponsive to traditional treatments, underwent treatment with intravenous pamidronate, after providing a written consent from each patient. Lymphedema diagnosis was based on history, physical examination and was confirmed with radioisotope lymphangiography. Computed tomography and Doppler sonography were performed if there was any doubt about the diagnosis.

The patients were excluded if they had a metastatic disease, developing severe side effects or developed lymphedema after a surgical procedure. All patients had moderate to severe lymphedema without any elephantiasis (Miller’s stage П) 3 and with no renal insufficiency. It means that the edema was not spontaneously reversible by elevation or compression of the limb and a moderate to severe fibrosis was present. These patients had recalcitrant chronic lymphedema without any response to conservative management such as physiotherapy (manual drainage) or compression by pump, elastic sleeves and stockings.

All patients received calcium supplement before beginning of infusion if they had a normal or low calcium serum level. Disodium pamidronate concentrated solution (15 mg/ml; Wockhardt; UK) was administered intravenously once a month for 3 consecutive months (60 mg was diluted in 500 ml of dextrose or saline and infused during six hours). None of them had received other additional medications, such as steroids or benzopyrones for reduction of extremity edema. After receiving pamidronate, they continued their conservative treatment by elevation and compression stockings. Follow up examinations of patients was performed once a month during the treatment course and 3 months after by the same physician.

They were asked about side effects of drug (such as headache, myalgia and fever) and the relief of related previous complaints (such as limb heaviness, pressure sensation and limitation of movement). Objective evaluation by physician was also included measuring the discomfort by visual analog scale (VAS) and measuring of patient’s limb volume, and limb circumference. Patient’s limb volume was measured at each examination by water volumetry with using a measured tank and their lower limb circumference was measured at eight points with 10 cm intervals from the medial maleous to upper thigh. The Quantitative data from repeated measurements of limb volume and circumference were analyzed using SPSS software (Version 15, Chicago, IL, USA).

## Results

**Fig. 1:**
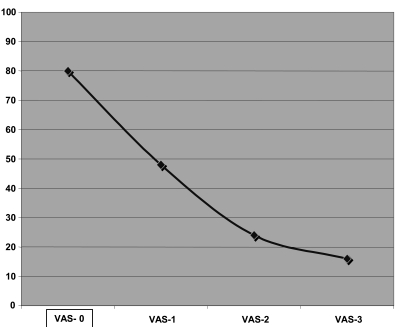
Mean Visual Analogue Score after each infusion of sodium Pamidronate (VAS:Visual Analogue Score ,each one months apart)

Among 12 patients, five were men and seven were women with an average age of 38.4 years (range: 24-61 years) received 3 consecutive courses of pamidronate. Table 1 summarizes the patient characteristics including age, sex, site, duration and etiology of the disease.Before treatment, all patients complained from lower limb edema (lymphedema) affecting their normal life and activity but after treatment with pamidronate, most of them felt much well and they could do their daily activities much better. Table 2 shows the baseline and subsequent measurements that were carried out for patients demonstrating a pretreatment median VAS score of 80 and a statistically significant change after the first infusion and a decrease to 16 after 3 months treatment with pamidronate (p=0.001) (Figure 1).

**Table 1:** Characteristics of 12 lymphedema patients treated with pamidronate

**Patient **	**Sex**	**Age**	**Site **	**Aetiology**	**Disease duration (Months)**
1	F	38	LLE^a^	Idiopathic	18
2	M	45	RLE^b^	Idiopathic	12
3	F	61	LLE	Idiopathic	10
4	F	49	LUE^c^	Idiopathic	8
5	F	38	LLE	Idiopathic	12
6	M	36	LLE	Idiopathic	10
7	M	45	LLE	Idiopathic	15
8	F	39	LLE	Idiopathic	11
9	M	24	LLE	Idiopathic	9
10	F	29	LLE	Idiopathic	14
11	F	36	LLE	Idiopathic	16
12	M	47	LLE	Idiopathic	7

^a^ LLE, Left Lower Extremity;

^b^ RLE, Right Lower Extremity;

^c^ LUE, Left Upper Extremity

**Table 2:** Changes of limb volume, circumferences and discomfort (VAS) after pamidronate infusions

	**Mean volume^a^(Liter)**	**Mean circumferences^a^ (cm)**	**VAS^b^ (0-100 mm)**
Baseline	4.56±0.8	39.5±9.7	80±12
1 st infusion	4.48±0.9	38.8±4.9	48±16
2 nd infusion	4.37±1.1	36.5±4.5	24±13
3 rd infusion	4.12±1.2	35.3±3.1	16± 5
Differences	-0.44	-4.2	-64

^a^ Measurements one month after infusion

^b^ Pain and discomfort one month after infusion.

The limb volume progressively decreased in affected limb with each infusion of the drug and the mean lower limb circumference reduced in all measured points following each infusion of pamidronate. Except short time myalgia, no other serious side effect was noticed during or after administration of pamidronate. The data showed a considerable improvement of the lymphedema following treatment with pamidronate, specially the subjective satisfaction of the patients and the functional improvement of them.

## Discussion

Extremity lymphedema may cause great discomfort for the patient and most of the cases inadequately responded to the treatment, thus efforts to increase patients’ comfort are greatly needed. This case series are the first report of pamidronate use for lymphedema which was added to management of the patients due to inadequate response to conservative managements such as limb elevation and compression therapy. The results showed that pamidronate had incredible effects on lymphedema, with improved patient’s comfort, increased functional capacity, and reduced their limb volume. We did not find any predictive factor for response to pamidronate. It is well tolerated without any life threatening side effect in these patients. Some of possible speculations on the mechanism of action of pamidronate in lymphedema could be a reduction of vasodilation, an inhibitory effect on the afferent nerves and the neuropeptide release.[24] These effects could explain both pain and reduction in limb edema with interaction with the microcirculation, tissue trophism, etc.

The absence of a control group was the limitation of our study but it provides an objective evidence for the effectiveness of pamidronate to be well tolerated without any life threatening side effect. However, the need for an intravenous use and reduction of cost are a matter of concern. Our study may provide a new therapeutic option to improve the quality of life of patients suffering from refractory lymphedema. However, further randomized, double blind, controlled placebo investigations are necessary to confirm these benefits.
